# Impact of Information based Classification on Network Epidemics

**DOI:** 10.1038/srep28289

**Published:** 2016-06-22

**Authors:** Bimal Kumar Mishra, Kaushik Haldar, Durgesh Nandini Sinha

**Affiliations:** 1 Department of Mathematics, Birla Institute of Technology, Mesra, Ranchi, 835215 India; 2 Department of Mathematics, Birla Institute of Technology, Mesra, Ranchi, 835215 India; 3 Adjunct Assistant Professor, Department of Mathematics, Temple University, Philadelphia, USA.

## Abstract

Formulating mathematical models for accurate approximation of malicious propagation in a network is a difficult process because of our inherent lack of understanding of several underlying physical processes that intrinsically characterize the broader picture. The aim of this paper is to understand the impact of available information in the control of malicious network epidemics. A 1-n-n-1 type differential epidemic model is proposed, where the differentiality allows a symptom based classification. This is the first such attempt to add such a classification into the existing epidemic framework. The model is incorporated into a five class system called the DifEpGoss architecture. Analysis reveals an epidemic threshold, based on which the long-term behavior of the system is analyzed. In this work three real network datasets with 22002, 22469 and 22607 undirected edges respectively, are used. The datasets show that classification based prevention given in the model can have a good role in containing network epidemics. Further simulation based experiments are used with a three category classification of attack and defense strengths, which allows us to consider 27 different possibilities. These experiments further corroborate the utility of the proposed model. The paper concludes with several interesting results.

Scientific efforts to model and accurately approximate the spread of malicious content over the Internet have received significant attention from researchers ever since the appearance of the Morris worm in 1988. The aims and methods employed by attackers, as well as the level of damage inflicted, have all changed significantly, over the years. The aim initially was to infect as many computers as possible. Then the infected computers were used to spread the infection in an automated manner with an exponential rate of spread^1^. This characterized the so-called *fast spreading worms*, which included the highly popular Code Red^2^ and Nimda worms^3^. Internet security threats have evolved consistently and considerably over the last one and a half decade as the malware authors have shown constant innovation in their methodologies. Recently the number of attacks which focus primarily on a finite, often small, set of specific IP addresses has started to gain significant popularity. Such attacks are called *targeted attacks.* Symantec reports reveal an ever increasing trend in the global average of reported cases of targeted attacks since 2010. The reported number was 77 in 2010, 82 in 2011^4^, and then 116 in 2012^5^. These attacks are characterized by malicious intentions like cyber espionage (Ghostnet attack 2009), cyber sabotage of critical physical resources (Stuxnet attack of 2010), and industrial espionage (Nitro attack of 2011). The traditional threats are now becoming more critical as they are expanding into newer forums like social media and mobile devices. The proportion of mobile malware has also shown a steadily increasing trend over the last few years. Symantec reported a 58% increase in the number of mobile malware families in 2012^5^.

The constant use of specialized techniques for intrusion and also customized tools makes it very difficult to defend against such attacks. Stealth techniques incorporating patience and persistence are being used to reduce the detection risk. In the light of such attacking methods, the traditional manual patching approaches to defense are clearly not efficient. The need is to develop detection and response systems which are intelligent enough to identify malicious attacks before they are able to inflict serious damage. The decision making can be improved by including a cooperative strategy where appropriate additional information about the status of infection is readily available. Such information may include data on the infection rate, or the response rates of different nodes in the network. This collective approach can help in a meaningful use of the available evidence on the severity and certainty of an attack, which is rarely used.

There are several approaches that have been suggested over the years for a proper understanding of malware and their spread on networks. The initial theory based models proposed by the founding fathers of this domain are responsible for giving birth to what is now called theoretical computer virology^6^,^7^. Their methods were based on the intriguing similarities that existed between viruses which are computer based and those that are biological. A very novel suggestion was made by Murray when he highlighted that the methods existing for the study of epidemic spread of biological infections could be useful in understanding the propagation of computer viruses^8^. A popular biological epidemic model called the SIS (Susceptible - Infectious - Susceptible) model was then applied as the first such application to study the manner in which computer viruses spread on different kinds of networks^9^. The approaches may roughly be classified into two broad categories. In the first category, we can put those approaches which are based on purely epidemic homogeneous contact models^10^,^11^,^12^. Such models are devoid of the complexities arising from topological considerations. They are also robust enough in providing strong analytical insights about various dynamical properties of the system like epidemic thresholds, equilibrium points of the system, stability of the equilibria, and periodic behavior of solutions, among others. In the second category are included approaches that rely on the topology of networks. Such approaches have provided useful results on the existence of epidemic thresholds for simple models including the SI, SIS and SIR models^13^,^14^. There is however a difficulty in proving theoretical results like stability of equilibrium points, owing to the large number of different kinds of possible topologies of large scale networks. Instead of theoretical proofs, often simulation and experimentation based proofs have been provided. One of the most important findings of this category of approaches is the lack of the universal epidemic threshold for infinite-size scale-free networks^15^. Another important contribution was the N-intertwined mean-field approximation based model and its fully heterogeneous extension^16^,^17^. These models provided useful dimensions outlining the relation between network topology and the spreading process on the network. In the epidemic approaches used so far, there has still not been an effort to include the effect of anticipating such attacks before they actually occur. Instead of a *wait-and-watch* approach, anticipation of an epidemic path can be useful in identifying the course of action to pursue.

This paper basically addresses the following research scopes:

- How can we model the spread of an attack in a network with respect to time? How does a network attack start from one or two nodes and propagates to infect often millions of nodes?
- What is the long term behavior of the network with respect to time? A network may recover completely in most scenarios, but is there a possibility that a number of nodes remain infected? If so, what is the stable value of such a fraction of infection that persists?
- Is there a threshold condition that determines the long term behavior of the system with respect to the infection persisting or perishing? Such a threshold exists in epidemic literature and is called the *basic reproduction number*. Based on related ideas, we try to obtain a threshold condition for our system as well.
- Can the symptoms exhibited by nodes infected by different types of attacking agents be used to improve the intelligence level of the underlying detection and response system? What is the impact of such a behavioral classification on the spread of infection? Based on the above classification, can the network be made to react in a more efficient manner?
- Is there a possibility that the nodes use the additional information available with them and also disseminate it, so that they can act in a collaborative manner?

The remaining portion of the paper is structured as follows. The proposed architecture is presented in the next section. Then the various aspects of the model and its mathematical formulation are detailed. The next section analyzes the model to establish the long term behavior of the epidemic system. Experiments and the corresponding results are then discussed. Finally, the paper is concluded with an elaboration on the major findings of the present work.

## Proposed Architecture

**Figure 1:**
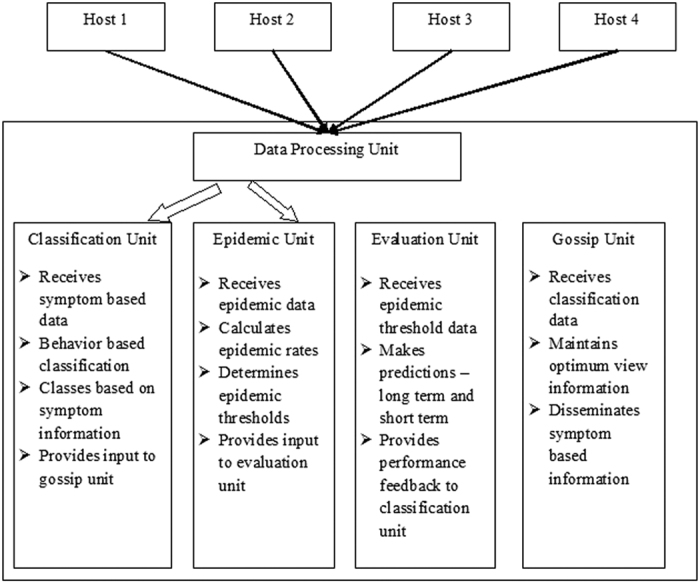
DifEpGos Architecture.

The proposed architecture for a differential symptom based epidemic classification and defense is shown in Fig. 1. The architecture involves five different components which are as follows:

### Data Processing Unit

This component receives raw data from the different hosts and extracts two kinds of information necessary for the working of the other components. It uses the raw data to get meaningful information that is subsequently used for a behavioral classification. This information is sent as an output to the classification unit. It also extracts the epidemic data from the raw data and sends it as an output to the epidemic unit.

### Classification Unit

This component uses the behavior based data received to perform a behavioral classification. A number of classifiers exist for an automated malware classification and analysis. The work of Bailey *et al*. can in particular be mentioned^18^. They first examined the effectiveness of existing host based antivirus products in providing semantically meaningful information concerning the malicious software (or malware) and tools used by attackers. Using a large collection of malware that spanned a variety of attack vectors, it was shown that different antivirus products characterize malware in different ways. This characterization is inconsistent across antivirus products, incomplete across malware, and they fail to be concise in their semantics.

They proposed a new classification method that described malware behavior in terms of number of system state changes like files written, processes created, etc. and not in sequences or patterns of system calls. Also to address the large volume of malware and the diversity of their behavior, a method was provided to automatically categorize these profiles of malware into groups representing similar classes of behaviors. They also demonstrated how behavior based clustering provides a more direct and efficient way of classifying and analyzing Internet malware. In the present paper, we do not attempt to go into the details of the classification unit and the relative efficiency of the classification algorithms that can be used, but it can be taken up as a separate work. The information regarding the number of classes, and the symptoms associated for the classification, and the corresponding optimal defense mechanism is sent as an output to the gossip unit.

### Epidemic Unit

This component receives the epidemic data from the data processing unit and then uses it to find a number of values, which can be used to effectively describe the epidemic state of the whole network. These values will be in the form of a number of rates and also an epidemic threshold. Subsequent sections of this paper focus on finding these values, and establishing their relevance. Its output is sent to the evaluation unit.

### Evaluation Unit

This component uses the data received by it to perform short and long term predictions regarding the epidemic state of the system. This basically allows evaluating the performance of the system, and particularly the classifier involved. A negative feedback may be used as a suggestion to fine tune the performance of the classification unit.

### Gossip Unit

This component plays the essential role of disseminating the classification information to the hosts. It also needs to optimize the view that it chooses to use. An efficient working of this unit is important mainly because it is responsible for providing the backup support needed for an intelligent anticipation by the overall system.

The epidemic information needs to be analyzed, for which a suitable epidemic model is necessary. A model incorporating the difference in symptoms is proposed in the subsequent sections. We call our architecture as *DifEpGoss* architecture as it is based on such an *EPidemic* model which uses a *DIF*ference in symptoms, along with a *GOss*ip based information dissemination.

## The D-SEIR Model and its mathematical formulation

**Figure 2:**
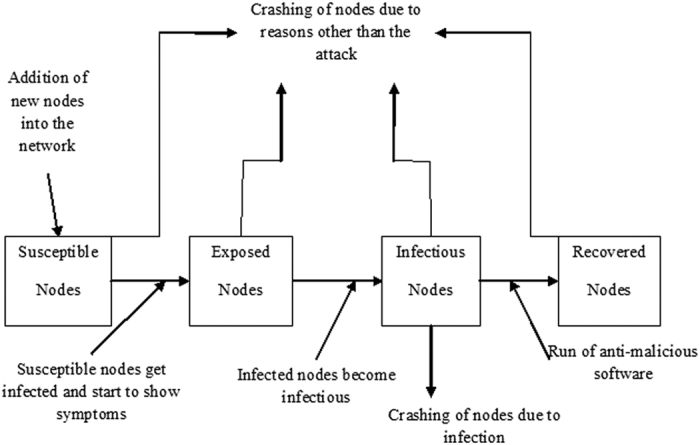
The SEIR (Susceptible-Exposed-Infectious-Recovered) framework.

In this paper, we use the *Susceptible-Exposed-Infectious-Recovered* (SEIR) model^12^,^19^ as the basic underlying framework. Figure 2 provides a schematic representation of this model. We attempt to provide greater significance to the model, by making use of the fact that once a system gets infected and it starts to show specific symptoms, then the role of the defense mechanism can be more targeted and based on intelligent anticipation. If there is a specific response to the stage where identified symptoms are just beginning to appear, then there is a greater chance that even a strong attack can be thwarted before it becomes significant. We do not attempt to make a classification of specific symptoms or the specific defense to be adopted, but use a more abstract approach and consider n different groups or sub-classes based on the symptoms exhibited. The proposed model considers a difference between nodes based on the characteristic features or symptoms exhibited, and hence it is referred hereafter as the *differential – SEIR* or D-SEIR model. The assumptions that lead to a formulation of the model can be enlisted as follows:

### Initial susceptibility

All nodes in the network are initially taken in the susceptible (S) class. This accounts for the fact that the modeling process starts at time zero for an attack. All nodes are thus non-infected at that point but have a chance of being infected, as time progresses.

### Differential Infection probability

It is assumed that the probability of the susceptible nodes getting infected into the i^th^ exposed sub-class (E_i_) is 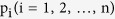, such that 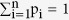. This assumption allows mathematical tractability but a point that arises out of it is whether it is essential for all nodes to become exposed, before getting recovered. This point can be included by introducing the concept of direct immunity. In the present paper, this factor has been ignored for the sake of simplicity, but it can very well be included for a more concrete analysis in some related future work.

### Node removal from network

The removal of nodes from the network is assumed to be because of two reasons. Firstly when nodes succumb to the infection (at a rate δ), and secondly due to node failures for other reasons (at a rate μ). Other reasons may include hardware failure, physical damage, or power discharge (in case of sensor and ad hoc networks). Such kind of removal can occur from each of the four classes. Removal because of infection however takes place only from the infectious (I_j_) sub-classes.

### Post latent infection

A latently infected node in sub-class *E*_*i*_ becomes infectious and moves into sub-class I_j_ with a rate *γ*_*ij*_. One fact that needs to be accounted for is that it is not at all necessary that the transitions occur only between the corresponding sub-classes (i.e. *E*_*i*_ to *I*_*i*_ only). There is a possibility that a symptom is misclassified or a node shows symptoms of more than one class. Under such a scenario, the probabilities for *E*_*i*_ to *I*_*j*_ transitions will have non-zero value. The situation where all probabilities except the corresponding ones are zero (i.e. only *E*_*i*_ to *I*_*i*_ probabilities exist) will be possible only when we have an *ideal classifier* with no classification error. In the present model, therefore small non-zero values have been assumed for *E*_*i*_ to *I*_*j*_ transitions (i ≠ j) and for transitions between the corresponding sub-classes (when correct classification is made) higher values have been assumed.

### Recovery

The infectious nodes get disinfected on use of anti-malicious measures. Upon recovery the nodes from each of the infectious sub-classes (*I*_*j*_) move into the recovered class (R). The immunity is considered to be permanent based on assumptions already specified earlier (in case of the SEIR model).

### Contact distribution

The average number of contacts per node is assumed as a function c(N) of the population size, i.e. *c*(*N*) = *c*_0_*N*, where N is the total population size and *c*_0_ is a constant of proportionality. This fits well into our homogeneity assumption. Here the nodes constituting the network are assumed to have an ability to interact and spread their infection to every other node, which is the most general form of interaction possible. The constant c_0_ is the factor by which the number of contacts scales as the population of the network varies. It basically provides a best case situation for the malware to spread, and hence a worst case for the analysis. The distribution may be modified to fit in to other specific topologies.

**Figure 3:**
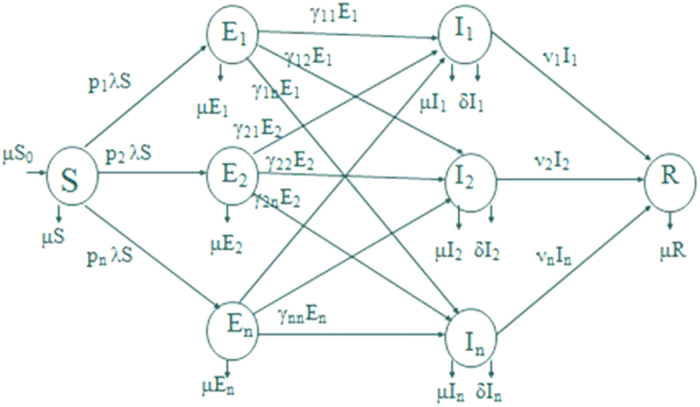
Schematic Representation of the D-SEIR Model.

Based on these assumptions, the model can be schematically represented as in Fig. 3 below.

The nomenclature of basic terminology used in the model is summarized in Table 1.

The transformations shown in Fig. 3 can be used to obtain the following system of ordinary differential equations, which gives the mathematical representation of the model.


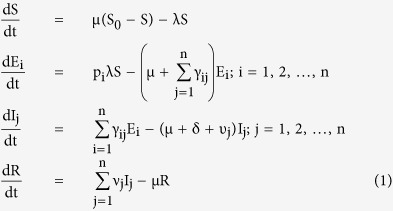


where the total population size is


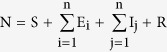


considering the average number of contacts per node c(N) as a function of the population size and β_j_ as the infectivity of nodes in j^th^ infectious class, the rate of infection λ for the nodes in the susceptible class can be given as


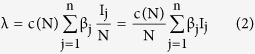


here 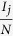 represents the probability that a contact with a node of infectious sub-class j results in an infection. Assuming *c*(*N*) as being directly proportional to the population size N, we have *c*(*N*) = *c*_0_*N*, and so the rate of infection reduces to a bilinear form, given as


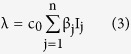


The rate of infection is thus dependent on the total sum of infectivity of nodes, where we consider the infectivity of the corresponding infectious sub-class. In the next section, the various analytical aspects of the model are discussed.

## Stability Analysis

In this section our focus is on examining the long term behavior of the network with respect to time. The model is analyzed to find conditions under which the network will recover completely or if there is a possibility that a number of nodes will remain infected. In such a case, the stable value of the persisting infectious fraction will also be found. Firstly, we establish an epidemic threshold. It will determine the conditions for long term behavior of the system and would enable us to know if the infection persists or dies out.

### Epidemic threshold

The epidemic threshold will be a value *R*_0_ called the *basic reproduction number* (borrowing terminology from biological epidemics) which may be defined as follows.

**Definition 1 (Basic Reproduction Number).** The basic reproduction number (*R*_0_) may be defined as the expected number of secondary infections produced by a single node during its entire infectious period, in a population of all susceptible nodes^20^.

The value of *R*_0_ will be used to obtain an epidemic threshold (say *τ*_0_) which is a value such that

- the infection dies out over time if *R*_0_ < *τ*_0_
- the infection persists and becomes an endemic if *R*_0_ < *τ*_0_

We first obtain a value of *R*_0_ for our model and then use it to find conditions involving the epidemic threshold.

**Theorem 1.** The value of the basic reproduction number for the D-SEIR model is given as


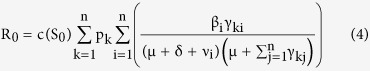


*Proof*. The derivation follows along a method called the *next generation matrix* method^20^,^21^,^22^.

A quantity that would be useful in the derivation is the partial derivative of the infection rate *λ* at the infection free equilibrium (IFE), which is given as


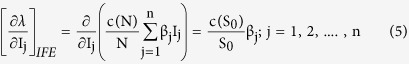


The Jacobian at the infection free equilibrium is given as


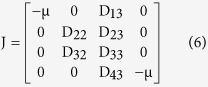


where the elements of the block matrix J are given as follows


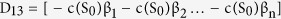



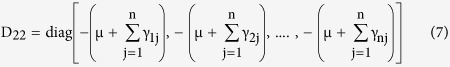



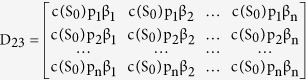



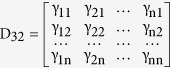



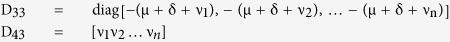


We consider the system of equations with the infected classes represented first, and from it we obtain the matrices representing the rate of appearance of new infections (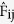) and the matrices representing the difference between outward and inward flow of nodes into a compartment (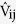) as follows


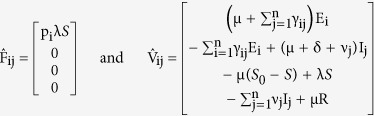


Next, taking the partial derivatives with respect to the infectious classes, we get


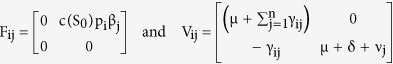


and then generalizing we have,


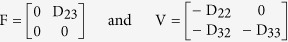


where the sub-matrices are as defined in (7), and the two matrices are observed to be non-negative and non-singular respectively. Now the basic reproduction number is given as the spectral radius (ρ) of the next generation operator FV^−1^ and so we have


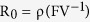


where, the inverse of the block matrix V is given as 
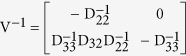


So, the value of R_0_ becomes


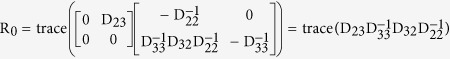


whose diagonal elements are 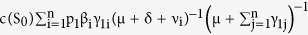,


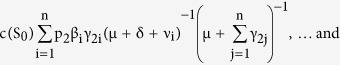



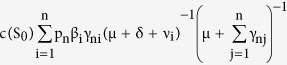


and hence we have


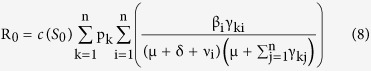


◽

### Equilibrium Points

For a system of differential equations, an equilibrium point (also called critical point or equilibrium solution) may be defined as follows:

**Definition 2 (Equilibrium Point).** For a system of differential equations


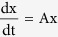


a substitution of zero in the right hand side gives points that correspond to constant solutions (that do not change with time) and are called equilibrium points^23^.

The D-SEIR model has two equilibrium points. The first of these has a zero value for all *I*_*j*_ (and in fact for all *E*_*i*_ as well) and hence is referred to as the *infection free equilibrium* (IFE) point. The second one, on the other hand, has a positive component of infection and hence is called an *endemic equilibrium* point.

**Infection free equilibrium:** The infection free equilibrium point for the D-SEIR model is given as 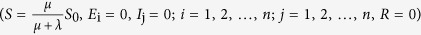


**Endemic equilibrium:** The endemic equilibrium point for the D-SEIR model is given as


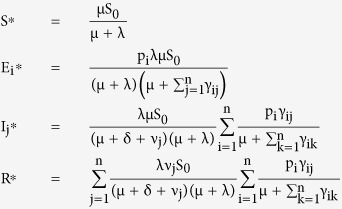


where


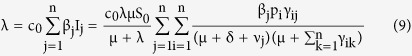


The relevance of the endemic equilibrium point is that it gives a quantitative measure for the infected population, when the infection survives. This allows us to have an estimate for the number of nodes that are expected to be infected in the long run.

### Value of the threshold *τ*_0_

The definition of the basic reproduction number as the expected number of secondary infections induced by a single infected host, leads us to an intuitive idea the threshold has to be one. This is because when an infected node infects at least one other node, then only we can expect the infection to spread. In the following we give a mathematical reasoning for this intuitive idea.

We consider a function f to be defined as


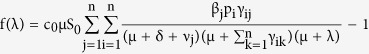


where λ is given by (9). Then the endemic equilibrium exists if and only if there exists a positive solution of 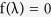.

Now its derivative with respect to λ is 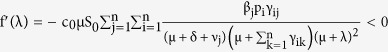


Also *f*(*λ*) → −1 *as λ* → ∞ and further 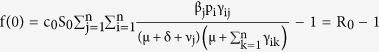


Therefore, a solution exists for f(*λ*) = 0, and hence an endemic equilibrium exists if and only if *f*(0) > 0 or *R*_0_ 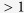.

### Stability of the Infection Free Equilibrium

The infection free equilibrium, as explained above, corresponds to a state where the infection disappears in the long run from the network. The epidemic threshold condition for this scenario was already seen to be *R*_0_ < *τ*_0_ and we also established the value of *τ*_0_ to be one. Next we consider the impact of a small or a large perturbation on the stability of the infection free equilibrium point. Stability on the face of a large perturbation will give us a guarantee that the infection will continue to disappear, even if the attack uses a large number of nodes initially. We thus consider the impact of a minor attack (corresponding to a small perturbation – referred to as local stability) and that of a major attack (corresponding to a large perturbation – referred to as global stability) on the stability of the infection free equilibrium. A mathematical proof is provided for the more stronger case of global stability.

**Theorem 2.** The infection free equilibrium is globally asymptotically stable when *R*_0_ < 1.

*Proof*. Here we prove the global stability of the infection free equilibrium (IFE) using Lyapunov’s method. For the total collection of nodes, we have


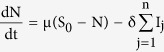


and so, 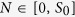. Then the domain Γ = {(S, E, I, R) | 0 ≤ N ≤ S_0_} where, E = (E_1_, E_2_, …., E_n_)^T^ and I = (I_1_, I_2_, …., I_n_)^T^, is positive time-invariant set for the system (1). A real-valued function L defined on Γ is selected, which is analogous to the potential function of classical dynamics, which is popularly referred to as the *Lyapunov function*. The function needs to have a non-negative value at all points in tis domain, and for stability at an equilibrium point it needs to have a zero value there and its time derivative at nearby points needs to be negative. This corresponds to the energy of a system which will dissipate as it approaches an equilibrium point. The choice of L considers the transitions for the infectious classes, and in general the i^th^ exposed sub-class and the j^th^ infectious sub-class has been taken into consideration. We consider the function


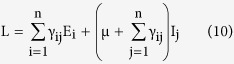


Then at the infection free equilibrium *L*(*IFE*) = 0 and otherwise 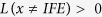, for all x ∈ Γ. Also, the time derivative of L is given as


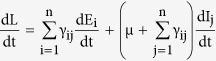


which on substitution of values of the derivatives from (1) becomes


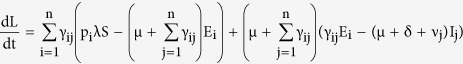


On simplification and cancellation of common terms and also using the value of the rate of infection λ, the equation reduces to the following form


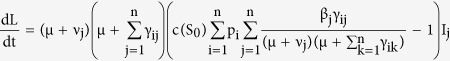


where the dummy index in the denominator has been changed to avoid repetition. Replacing the indices i, j and k inside the parenthesis with k, i and j respectively, the expression becomes


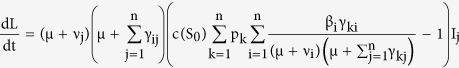


Using the value of R_0_ from (8), gives


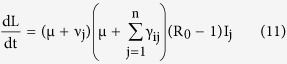


hence *dL*/*dt* < 0 if R_0_ < 1 and *dL*/*dt* = 0 if and only if R_0_ = 1 or *Ij* = 0, i.e. at the infection free equilibrium. As such the function L is positive definite over Γ and its time derivative is negative definite. Moreover when *R*_0_ > 1, then *dL*/*dt* > 0 if *Ij* > 0, which shows that the infection free equilibrium is unstable when R_0_ > 1. This validates the claim that the infection free equilibrium is globally asymptotically stable if R_0_ ≤ 1. ◽

Next we use numerical simulations on the D-SEIR model to illustrate the fact that the infection free equilibrium is both locally and globally stable (asymptotic stability follows because the trajectories approach the equilibrium point for large values of time).

**Figure 4:**
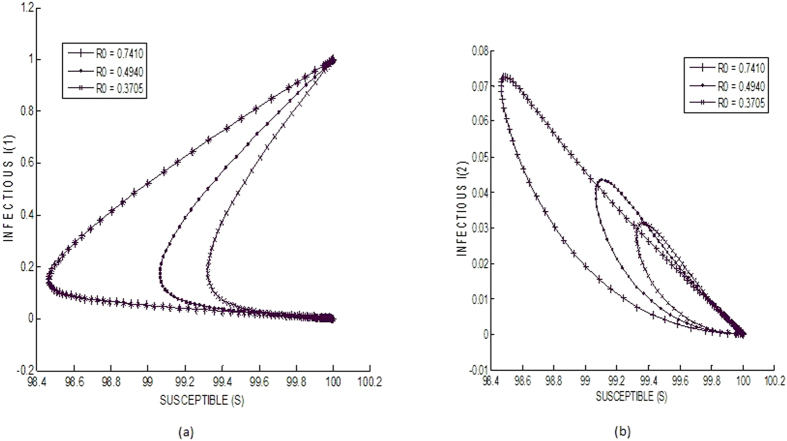
Local stability of infection free equilibrium.

Starting with an initial population of 100 susceptible nodes and 1 *I*_1_ node (for small perturbation – minor attack), the system is seen to stabilize to the infection free equilibrium (Fig. 4). Three different values of *R*_0_ have been used (0.7410, 0.4940 and 0.3705), all of which do not violate the threshold condition of *R*_0_ ≤ 1. In all the simulated cases, a total of three sub-classes have been considered for both the exposed (*E*_1_, *E*_2_, *E*_3_) as well as infectious (*I*_1_, *I*_2_, *I*_3_) classes. In Fig. 4, the asymptotic behaviors of the sub-classes *I*_1_ and *I*_2_ are shown. A difference is observed in the two sets of curves, owing only to the fact that they have a different initial infectious value (initially *I*_1_ is 1 and *I*_2_ is zero). Behaviorally they are similar behavior because in both cases the final value of *I* (i.e. *I*_1_ and *I*_2_) are both zero.

**Figure 5:**
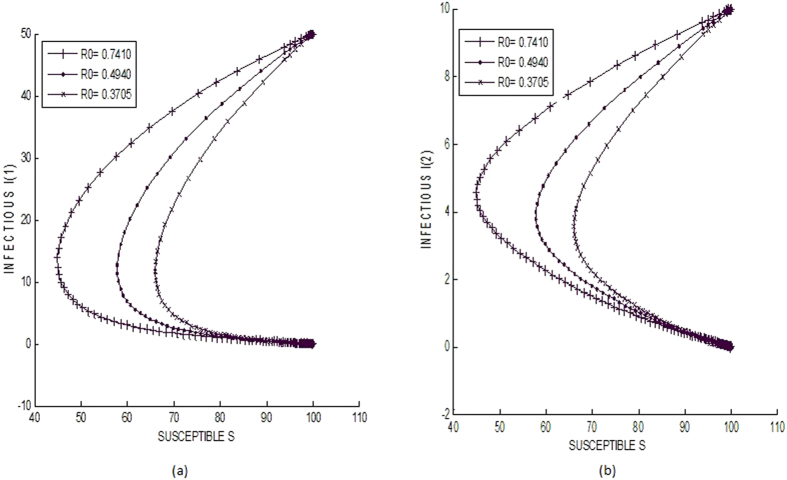
Global stability of infection free equilibrium.

Next we consider 50 *I*_1_ nodes initially and 10 nodes each of *I*_2_ and *I*_3_ subclasses (large perturbation). In Fig. 5, both *I*_1_ and *I*_2_ populations are seen to converge to the infection free equilibrium point. This shows the global stability of the infection free equilibrium for R_0_ ≤ 1.

### Stability of Endemic Equilibrium

**Theorem 3.** The endemic equilibrium is globally stable when R_0_ > 1.

*Proof*. We use the geometric approach suggested by Li and Muldowney^24^ to prove the global stability condition for the endemic equilibrium point. Based on this approach, it is known that if the mapping 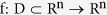, where D is an open set, be such that each solution x(t) of the differential equation x′ = f(x) is uniquely determined by its initial value x(0) = x_0_, and an equilibrium point 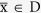 satisfies the following assumptions

(A1) D is simply connected

(A2) There is a compact absorbing set K ⊂ D

(A3) 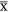 is the only equilibrium point in D,

then the global stability of 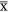 in D is given by the additional *Bendixson criteria*


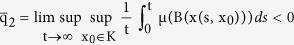


In this criteria, *x*(*t*, *x*_0_) denotes the solution *x*(*t*) determined by the initial point *x*_0_, and B is given as


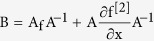


where 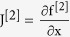
represents the second compound Jacobian matrix given as


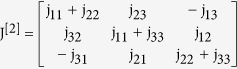


and A is a matrix-valued function satisfying


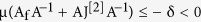


on K and μ denotes the Lozinskii measure, given as


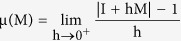


for an N × N matrix M.

Now the existence of a compact set that is absorbing in the interior of Γ follows from the uniform persistence of the system, where it can be shown that


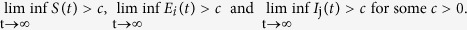


The proof for the Bendixson criteria 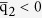, can be enumerated in the form of the following steps:

(1) Jacobian of reduced system: The reduced system obtained by neglecting the recovered class, which is possible because of its non-involvement in the dynamics of the other classes, is given as


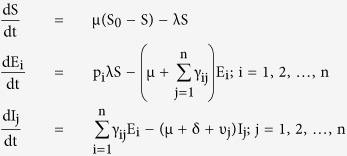


where 
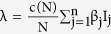


Then the Jacobian matrix of the reduced system is given as


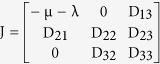


where


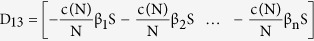



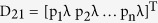



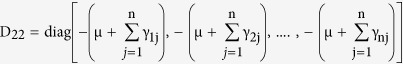



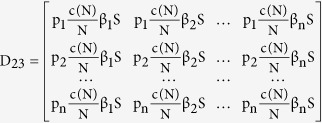



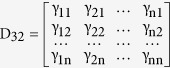



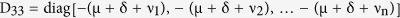


(2) Second Compound Matrix of the Jacobian: For the Jacobian matrix of the reduced system obtained above, the second additive compound matrix is given as


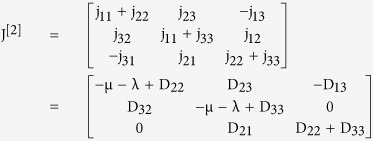


(3) Definition of matrix B in the Bendixson Criteria: We consider a diagonal matrix A defined as


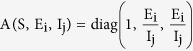


considering in general the i^th^ exposed sub-class and the j^th^ infectious sub-class. If f denotes the vector field of the system, then


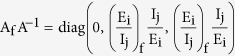


and


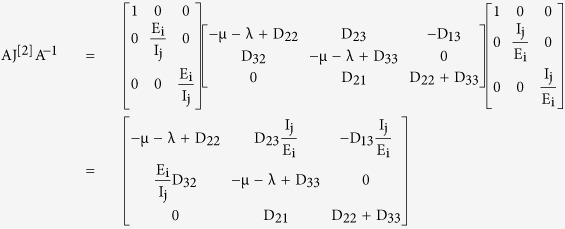


using these two matrices, gives the block matrix


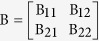


where


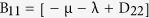



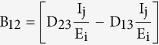



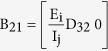



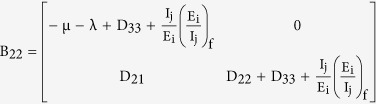


(4) Lozinskii Measure of matrix B: The Lozinskii measure for matrix B can be estimated as


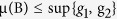


where g_1_ and g_2_ are defined as


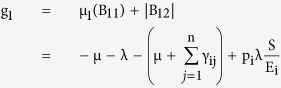


and,


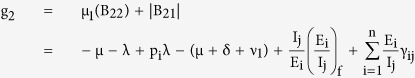


here the Lozinskii measure μ is with respect to the vector norm defined as


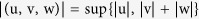


while the Lozinskii measure μ_1_ is with respect to the *l*_1_ norm and the norms of matrices B_12_ and B_21_ are also obtained with respect to the *l*_1_ vector norm.


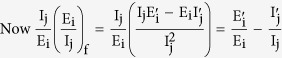


and so from the reduced set of equations,


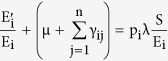


and


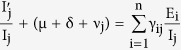


using these values in the equations for g_1_ and g_2_, gives


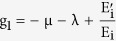


and


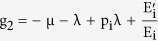


so, the Lozinskii measure of matrix B becomes


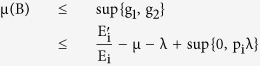


which finally gives


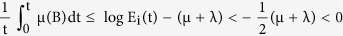


for all (S(0), E_i_(0), I_j_(0)) in the absorbing set, where the bound on the sizes of the classes are implied by the uniform persistence of the system.

Hence it is shown that the additional criterion 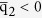 is also satisfied and thus the endemic equilibrium is globally stable. This condition also itself proves the local stability of the endemic equilibrium^24^. ◽

**Figure 6:**
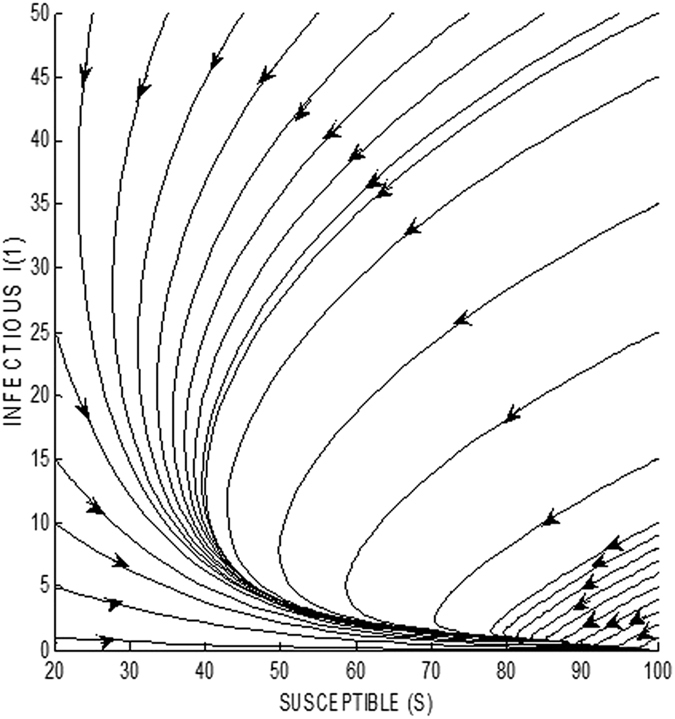
Global stability of endemic equilibrium point when R_0_ > 1 depicted in S − I_1_ phase plane.

Numerical simulations are once again used to clearly illustrate the situation in a phase plane. In Fig. 6, it is shown that for a value of R_0_ = 1.1116 (which exceeds the threshold value), there exists a stable endemic equilibrium point at (S* = 94.6992, E_1_* = 2.0738, E_2_* = 1.0371, E_3_* = 0.3456, I_1_* = 0.2592, I_2_* = 0.2304, I_3_* = 0.2034, R* = 0.4575). The figure shows the phase plane formed by the variables S (susceptible class) and *I*_1_ (first infectious sub-class). The trajectories are seen to asymptotically approach the stable endemic equilibrium point. The equilibrium point is unique and globally stable in the entire phase plane, as can be clearly seen.

**Figure 7:**
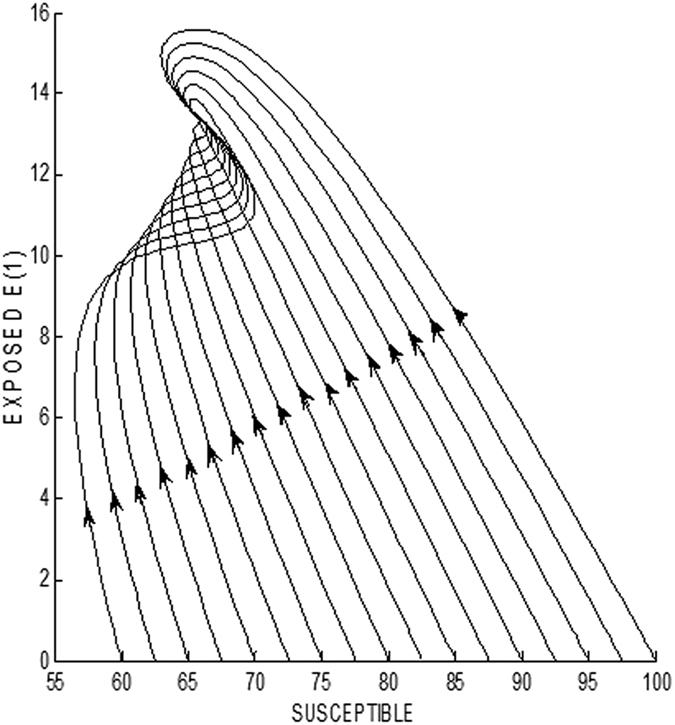
Global stability of endemic equilibrium point when R_0_ > 1 depicted in S − E_1_ phase plane.

In Fig. 7, the stability condition is verified using the phase plane formed by the variables S (susceptible class) and E_1_ (first exposed sub-class). In this case, the equilibrium point is observed to be (S* = 65.6816, E_1_* = 13.4289, E_2_* = 6.7145, E_3_* = 2.2378, I_1_* = 1.6786, I_2_* = 1.4921, I_3_* = 1.3166, R* = 2.9623) and can be seen to be globally asymptotically stable. Here, each of the trajectories assumes initially 10 infective nodes in the population.

In the next section, experiments are performed for both real and synthetic data to explore the validity of the proposed model.

## Numerical Simulations

In the previous section we obtained a threshold condition defined in terms of the basic reproduction number. Conditions for the infection to disappear with time or to persist into an endemic were also highlighted. Experiments through simulations were already used to verify the results as they were analytically obtained in section 4. In this section we perform some more experiments, mainly to bring forth the impact of the classification into sub-classes that was suggested in the model. We first perform the experiments with a real network dataset. It shows that the results can be generalized and applied to networks, with varying underlying topology.

### Real network data

The experiments on real network datasets which includes an AS (autonomous systems) graph instance containing AS-level connectivities inferred from the Oregon route-views. Three datasets are used- peer.oregon.010331, peer.oregon.010414 and peer.oregon.010505, which are available online at http://topology.eecs.umich.edu/data.html. The datasets contain pairs of interconnected ASs according to the Oregon route-views of a given collection date. The total number of undirected edges in the resulting AS graph for the three datasets were 22002, 22469 and 22607 respectively.

In the experiments three situations are explored:

- **No classification:** Spread of infection on the network without any classification (with an infection of every fifth susceptible node encountered and a recovery of one node after every 10 units of time).
- **No prevention:** Spread of infection on the network with classification into two kinds of attack (one with infection as earlier and the other for every susceptible node encountered; the recovery remains same).
- **D-SEIR model (with prevention):** Spread of infection on the network with classification and prevention of more severe attack (infection same; recovery of critical class is one node for every time unit, and for other class it is every fifth time unit).

**Figure 8:**
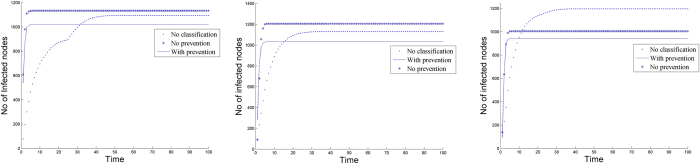
Performance of the D-SEIR model on the Oregon datasets.

The results are shown in Fig. 8. The third case is clearly seen to have the least infection value once the network stabilizes. For all the three datasets, initially 20 nodes were randomly selected to spread the infection. The asymptotic values were plotted in each of the cases.

### Simulative experiments

The experiments performed in this section are based on the following assumptions:

#### Attack and defence categories

Three abstract categories of attacks have been considered – strong, medium and mild. Analogously the defence mechanism is also considered to be of three types – strong, moderate and weak. Three classes have been assumed in the model for the exposed (E_1_, E_2_, E_3_) and infectious (I_1_, I_2_, I_3_) populations. It is also assumed that the first class in the model is equipped with a strong defence system, the second class with a moderate defence and the third class with a weak defence.

#### Variable parameters

**Figure 9:**
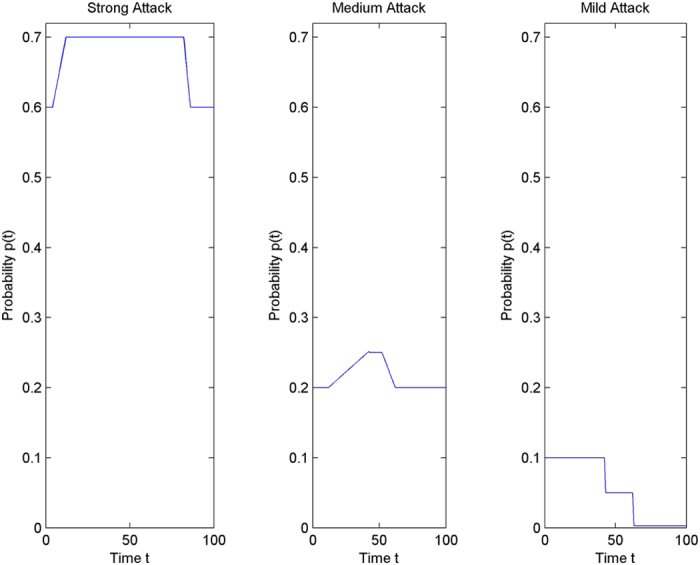
Characterization of Attack Categories.

The parameter values are assumed to be variable. This makes it possible to quantitatively analyze more realistic scenarios with respect to the interaction between the attacking and defence mechanisms. The attacking scenario is represented through a variable probability of infection (p_i_), whose characterization is as shown in Fig. 9. From the figure, it can be seen that:

- A strong attack takes effect instantaneously, i.e. it spreads at a very fast rate and infects as many systems, as quickly as possible. It has a large impact for a long duration. It then slowly starts to lose its effect, because of various reasons like network congestion, or inability of the scanning algorithm to further detect more vulnerable systems.
- A medium attack begins with a lesser impact compared to a strong attack. It then spreads but again to a comparatively smaller scale. It also takes greater time to reach its peak stage, and also spends relatively lesser time in this stage. Both the strong and medium attacks have been characterized using trapezoidal functions, but with different slopes, peak values and time spent at the peak.
- Mild attacks have been characterized using a two step decreasing function. This allows us to consider a very less starting impact, which subsequently becomes negligible.

**Figure 10:**
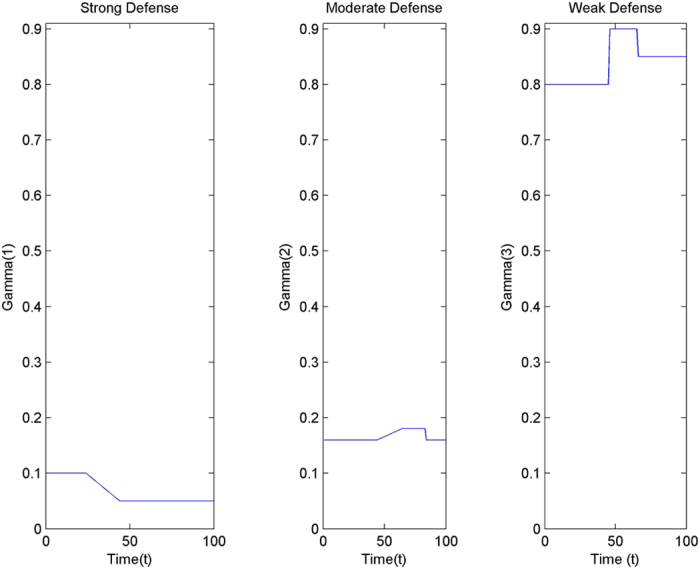
Characterization of Defense Categories

The characterization of the defense scenario is shown in Fig. 10. Here the modeled parameters are γ_i_ where the value i = 1 represents a strong defense, i = 2 represents a moderate defense while i = 3 represents a weak defense scenario.

#### Impact of D-SEIR model

**Figure 11:**
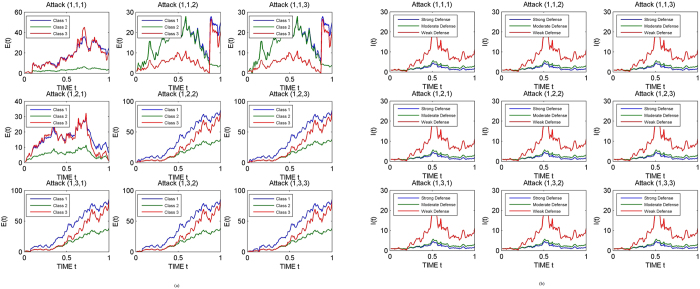
(**a**) Dynamical behavior of system for a strong attack in class 1 (**b**). Impact of Model on Dynamical Behavior of System for a Strong Attack in Class 1

Next we consider 27 possibilities arising from our consideration of 3 attack types and 3 defense types for 3 classes. This number will change depending on the actual considerations. In Fig. 11(a), a strong attack has been considered in the first class (shown by a 1 as the first element in the attack triplet). Consequently greater and faster spread of infection is observed in class 1. In Fig. 11(b), it can be seen that even a strong attack is not able to survive when the D-SEIR model uses a strong prevention.

It is thus clear that a distributed defense is able to change the course of even a very strong attack.

## Conclusion

Epidemic studies are known to provide important insights on network epidemics. Various kind of information may be obtained including the scale and long-term behavior of an attack. Epidemic models however still do not use available information to improve the model performance. In this paper, the utility of including available information in controlling the spread of a network epidemic was explored. A 1-n-n-1 type differential epidemic model has been proposed and analyzed to see the improvement in quality of an epidemic system. An overall epidemic architecture is also suggested that can be useful in providing a more practical utility to the epidemic models. An epidemic threshold of the system was obtained which clearly demarcated the long-term behavior of a network epidemic into two exhaustive classes, one with persistent infection and the other without any infection. An analysis of real network datasets also revealed a better performance for the model in controlling an epidemic when compared to previous models. Simulation based experiments allowed us to perform generalized scenario based experiments, which again corroborated the analytical findings. In future, the model can be extended to deal with different specific network topologies.

## Additional Information

**How to cite this article**: Mishra, B. K. *et al*. Impact of Information based Classification on Network Epidemics. *Sci. Rep.* **6**, 28289; doi: 10.1038/srep28289 (2016).

## Figures and Tables

**Table 1:** Nomenclature used in the Model.

Nomenclature			
S(t)	Number of nodes in the susceptible class.	n	The number of exposed and infectious sub-classes.
E_i_(t)	Number of nodes in the i^th^ exposed sub-class.	S_0_	Initial number of nodes in the network.
I_j_(t)	Number of nodes in the j^th^ infectious sub-class.	λ	Rate of infection of susceptible nodes.
R(t)	Number of nodes in the recovered class.	γ_ij_	Rate at which exposed nodes in the i^th^ subclass become infectious into the j^th^ subclass.
N(t)	Total number of nodes in the network.	δ	The per capita death rate due to infection.
μ	The per system death rate due to reasons other than the infection	ν_j_	Rate of recovery of infectious nodes in the j^th^ sub-class.
β_j_	Infectivity of nodes in j^th^ infectious sub-class.	*c(N)*	Average number of contacts per node.
